# Galunisertib (LY2157299), a transforming growth factor-β receptor I kinase inhibitor, attenuates acute pancreatitis in rats

**DOI:** 10.1590/1414-431X20165388

**Published:** 2016-08-08

**Authors:** X. Liu, M. Yu, Y. Chen, J. Zhang

**Affiliations:** 1 Department of General Surgery, the Affiliated Hospital of Qingdao University, Qingdao, China; 2 Department of General Surgery, People's Hospital of Chengyang, Qingdao, China; 3 Department of Clinical Laboratory, the Women and Children's Hospital of Qingdao, Qingdao, China; 4 Department of Traditional Chinese Medicine, the Affiliated Hospital of Qingdao University, Qingdao, China

**Keywords:** Acute pancreatitis, TGFβ1, NF-κB, Galunisertib

## Abstract

Galunisertib (LY2157299), a selective ATP-mimetic inhibitor of TGF-β receptor I (TGF-βRI), is the only known TGF-β pathway inhibitor. In the present study, we investigated the effect of galunisertib on taurocholate (TAC)-induced acute pancreatitis (AP) in rats, and the role of TGF-β and NF-κB signaling pathways. AP was induced by infusion of TAC into the pancreatic duct of Sprague-Dawley male rats (n=30). The rats (220±50 g) were administered galunisertib intragastrically [75 mg·kg^-1^·day^-1^ for 2 days (0 and 24 h)]. Serum IL-1β, IL-6, TNF-α, amylase (AMY), lipase (LIP), and myeloperoxidase (MPO) levels were measured by ELISA. NF-κB activity was detected by electrophoretic mobility shift assay (EMSA); NF-κBp65 and TGF-β1 proteins, as well as TGF-βRI and p-Smad2/3 proteins, were detected by western blot assay. Cell apoptosis was detected by TUNEL assay. H&E staining was used to evaluate the histopathological alterations of the pancreas. Galunisertib treatment attenuated the severity of AP and decreased the pancreatic histological score. In addition, number of apoptotic cells were significantly increased in the galunisertib-treated group (16.38±2.26) than in the AP group (8.14±1.27) and sham-operated group (1.82±0.73; P<0.05 and P<0.01, respectively). Galunisertib decreased the expression levels of TGF-βRI and p-Smad2/3 and inhibited NF-κB activation and p65 translocation compared with the sham-operated group. Furthermore, serum IL-1β, IL-6, TNF-α, AMY and LIP levels and tissue MPO activity were significantly decreased in the galunisertib-treated group. Our data demonstrate that galunisertib attenuates the severity of TAC-induced experimental AP in rats by inducing apoptosis in the pancreas, inhibiting the activation of TGF-β signals and NF-κB as well as the secretion of pro-inflammatory cytokines.

## Introduction

Acute pancreatitis (AP) is an inflammatory process in the pancreatic gland that may eventually lead to a severe systemic inflammatory response. A key event in pancreatic damage is the intracellular activation of NF-κB and zymogens, involving also calcium, cathepsins, pH disorders, autophagy, and cell death, particularly necrosis (1). The pathogenesis of AP is poorly understood. However, information from cellular and *in vivo* studies, as well as genetic studies in humans, suggests that pathological events that begin in the pancreatic acinar cell often initiate this disease (2).

Early events in AP lead to the activation of several pathophysiological mechanisms that result in local and systemic complications and organ failure, which is chiefly responsible for the mortality associated with the disease (3). The main mechanisms responsible for this systemic progression are pro-inflammatory cytokines, chemokines, reactive oxygen species (ROS), Ca^2+^, platelet activating factor, and adenosine, as well as neuronal and vascular responses (4). Furthermore, acinar cells can behave as inflammatory cells synthesizing and releasing cytokines, chemokines and adhesion molecules (5). Thus, acinar cells act jointly with leukocytes triggering the inflammatory response after the local damage of the pancreas. Pathological responses arising from the pancreatic acinar cells have a central role in initiating AP.

TGF-β is known to be active in almost every tissue and cell. Aberrant expression or dysregulated expression of TGF-β has been observed in various disease processes including autoimmune diseases, fibrosis and carcinogenesis (6). Recent studies have reported that TGF-β has a predominant role in the accumulation of pathological extracellular matrix in pancreatic fibrosis and chronic pancreatitis (7). Recently, numerous studies have found that TGF-β signal is activated in the early phase of AP, and inhibition of TGF-β signal decreases pathological injury to pancreas ([Bibr B08] [Bibr B09] [Bibr B10] [Bibr B11] [Bibr B12]–13), suggesting TGF-β signal could be a target for therapy of AP. Therefore, therapies aimed at reducing the impact of these activated factors during AP may be useful for preventing or treating AP.

Galunisertib (LY2157299), a selective ATP-mimetic inhibitor of TGF-βRI, is the only TGF-β pathway inhibitor currently under clinical investigation in hepatocellular carcinoma (HCC) and glioma patients (14). TGF-β1 protected NIH3T3 fibroblasts from Star-induced growth and mitochondrial damage. Additionally, several experimental studies found that LY2157299 could block TGF-β1 activation, resulting in cell growth inhibition and increased apoptosis ([Bibr B15] [Bibr B16]–17).

In the present study, we investigated the effect of galunisertib (LY2157299) on experimental AP and explored its mechanisms.

## Material and Methods

### Ethics

All animal studies were performed according to the Guide for the Care and Use of Laboratory Animals of the National Institutes of Health. The protocol was approved by the Ethics Committee of Animal Experiments of the Affiliated Hospital of Qingdao University.

### Agents

TGF-β1 and NF-kBp65 antibodies were purchased from Santa Cruz Biotechnology (China); p-Smad2, p-Smad3 and TGF-βRI antibodies were purchased from Cell Signaling (China).

### Preparation of acute pancreatitis animal model

Sprague-Dawley male rats (220±50 g, 10 rats per group) were maintained under controlled environmental conditions and fasted for 24 h with free access to water prior to experiments. AP was induced with 3% sodium taurocholate (TAC), by retrograde injection into the pancreatic duct as previously described (18). Briefly, rats were anesthetized with intraperitoneal sodium pentobarbital at a dose of 50 mg/kg. The abdomen was opened by midline incision to allow manipulation of the duodenum and biliopancreatic duct. The common bile duct was occluded, and the duodenal wall was punctured on the antimesenteric side with a 24-gauge catheter. The catheter was advanced into the papilla vateri and fixed to the duodenal wall. For inducing AP, the catheter was brought near the pancreatic canal and 3% TCA (0.1 mL/100 g; Sigma, USA) was infused slowly using a pump according to the retrograde ductal injection model, followed by closure of the abdomen in two layers. The same procedure was applied to the sham-operated group, to which 0.9% NaCl was administered instead of TCA.

### Galunisertib treatment

After the abdomen was closed the animals were allowed to recover for 2 h and then the AP group animals were administered galunisertib intragastrically (75 mg·kg^-1^·day^-1^) at 0 and 24 h. After 48 h, 0.25 ml blood samples were collected from the inferior vena cava and centrifuged at 10,000 *g* for 4–5 min to obtain serum and stored at −80°C until use for several assays. The animals were sacrificed by exsanguination while under ether anesthesia and the pancreatic tissues were rapidly collected for pathological examination and biochemical analyses. The same procedure was applied to the sham-operated group to which 0.9% NaCl was administered instead of galunisertib.

### Histopathological score

Pancreas was removed 48 h after the model was induced and treated with galunisertib or saline for morphological analyses, immediately immersed in 4% neutral phosphate-buffered paraformaldehyde for 12 h, embedded in paraffin, and cut into 5-μm thick sections, which were stained with H&E to observe the morphological changes under a light microscope. The severity of AP was blindly graded by a semi-quantitative assessment of vacuolization, edema, inflammatory cell infiltration and acinar cell necrosis according to a previous report (19) in ten microscopic fields, which were randomly chosen in each rat. Histological scoring of pancreatic tissue was performed to grade the extent of acinar cell vacuolization (0: none, 1: <20% acini with vacuoles, 2: <50% acini, 3: >50% acini), edema (0: no edema, 1: interlobular edema, 2: intralobular edema, 3: interacinar edema), inflammation (0: no inflammation, 1: interlobular inflammatory cells present, 2: intralobular inflammatory cells present, 3: interacinar inflammatory cells present) and acinar cell necrosis (0: no necrosis, 1: <10% necrosis, 2: <40% necrosis, 3: >40% necrosis).

### TUNEL assay

Cell apoptosis was analyzed using terminal deoxynucleotidyl transferase-mediated deoxyuridine triphosphate *in situ* nick end labeling (TUNEL) detection kit (Roche, China) following the manufacturer's instructions. Briefly, the tissue section was covered with equilibrium buffer for 5–10 min before addition of TdT reaction mixture. After incubation in the dark for 1 h, the tissue section was incubated with SSC solution for 15 min, followed by a final PBS wash. After DAPI counterstain, the tissue section was examined and photographed with a fluorescence microscope. Average number of fluorescence dots of three images from each treatment group was calculated.

### Electrophoretic mobility shift assay (EMSA)

Nuclear extracts from pancreatic tissues (100 mg) were prepared using a nuclear extraction kit, according to the manufacturer's instructions. Binding reactions consisted of 12.5 mM HEPES, pH 7.9, 50-100 mM NaCl, 5% glycerol, 2 mg/mL BSA, 2 μg poly-dIdC, 10 μg BSA, 0.1 mM EDTA, 0.1 mM DTT, 1 ng of 32P-end labeled double-stranded DNA probe, and 15 μg of nuclear protein. Binding reactions were incubated for 30 min at 21°C and then loaded onto 5% acrylamide-0.25X tris-borate-EDTA gels and electrophoresed at 200 V for 2 h. EMSA was carried out with consensus probes specific for NF-κB from Santa Cruz Biotechnology.

### Western blot analysis

Protein lysates were separated by 10% SDS-PAGE and transferred to nitrocellulose membranes. Following blocking with 5% non-fat milk in 1× Tris-buffered saline, pH 7.4, containing 0.05% Tween-20, the membranes were incubated with purified anti-NF-κB p65, TGF-β1, TGF-βRI, p-Smad2, and p-Smad3 antibodies at 4°C overnight. The following day, the membranes were washed with PBS and incubated with peroxidase-conjugated goat anti-rabbit IgG. Immuno-detection was conducted using chemiluminescence reagents and membrane was exposed on X-ray film. β-actin was used as an internal reference for relative quantification.

### Measurement of serum IL-1β, IL-6 and TNF-α levels

Serum IL-1β, IL-6, and TNF-α levels were measured using commercial enzyme-linked immunosorbent assay (ELISA) according to the manufacturer's instructions (B&C Co., China). All samples were tested in duplicate and are reported as the means.

### Serum amylase and lipase assays

Serum amylase (AMY) and lipase (LIP) levels were measured using standard techniques with a fully automatic chemistry analyzer (Olympus AU2700 Chemistry-Immuno Analyzer; Olympus Inc., Japan).

### Detection of myeloperoxidase (MPO) activity

The tissue samples were thawed, homogenized in 20 mM phosphate buffer, pH 7.4, and centrifuged at 10,000 *g* for 10 min at 4°C, and the resulting pellet was resuspended in 50 mM phosphate buffer, pH 6.0 (Sigma). The suspension was subjected to four cycles of freezing and thawing and further disruption by sonication (40 s). The sample was subsequently centrifuged at 10,000 *g* for 5 min at 4°C, and the supernatant was used for the MPO assay. This mixture was incubated at 37°C for 110 s; the reaction was terminated by adding 50 μL of 0.18 M H_2_SO_4_, and the absorbance was measured at 405 nm.

### Statistical analysis

Data are reported as means±SD. Statistical analyses were performed using SPSS software, version 17.0 (SPSS Inc., USA). The differences between the groups were analyzed using the Student's *t*-test or χ^2^ test. P<0.05 was considered to be a statistically significant difference.

## Results

### Galunisertib alleviated the histopathological alterations of the pancreas

**Figure 1:**
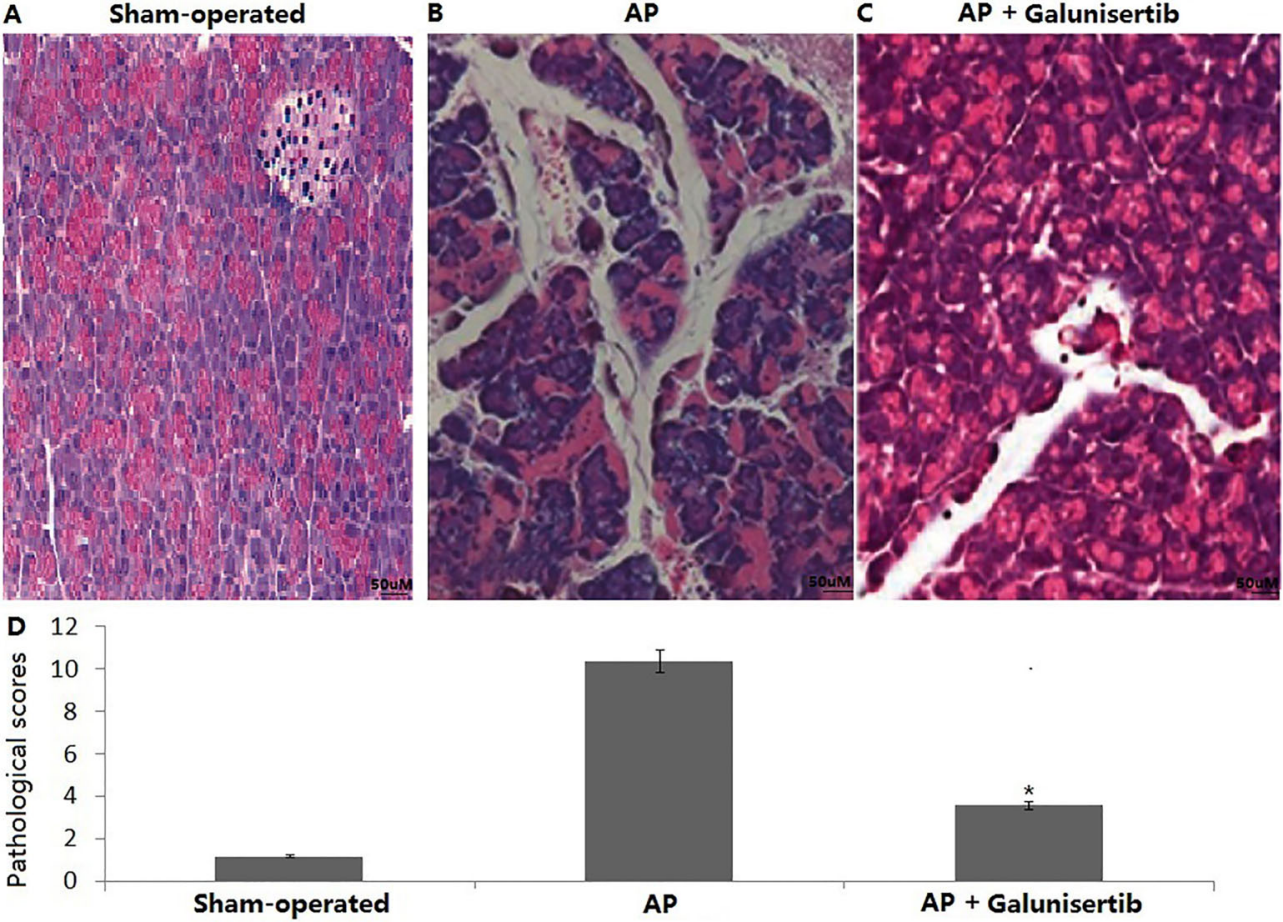
Pathological changes to the pancreatic tissues of the experimental groups in the acute pancreatitis (AP) model. Representative hematoxylin and eosin-stained sections were examined by light microscopy (original magnification, ×200). *A*, No obvious histological alterations were observed in the pancreatic tissues from the sham-operated rats; *B*, histological signs of pancreatic injury can be observed in the AP group; *C*, pretreatment with galunisertib showed significantly reduced injury in extent and severity; *D*, comparison of the total pathological scores of the pancreas in the three groups. Results are reported as means±SD (n=10 rats per group) *vs* the AP group, *P<0.05 (Student's *t*-test).

To detect the effects of galunisertib on the experimental animal AP model, the pathological changes in the pancreas of experimental groups were observed with an optical microscope. The sham-operated group showed normal pancreas (Figure 1A), whereas the AP group exhibited pancreatic changes indicated by acinar destruction, interstitial congestion, edema and inflammatory cell infiltration (Figure 1B). Rats in the galunisertib group showed significant improvement compared with the sham-operated group (Figure 1C).

In the AP group, the pathological score was 10.6±2.5, but in the galunisertib-treated group, the pathological score was 2.7±0.9, which was significantly lower compared to the AP group (Figure 1D, P<0.05).

### Galunisertib-induced acinar cell apoptosis of the pancreas

**Figure 2:**
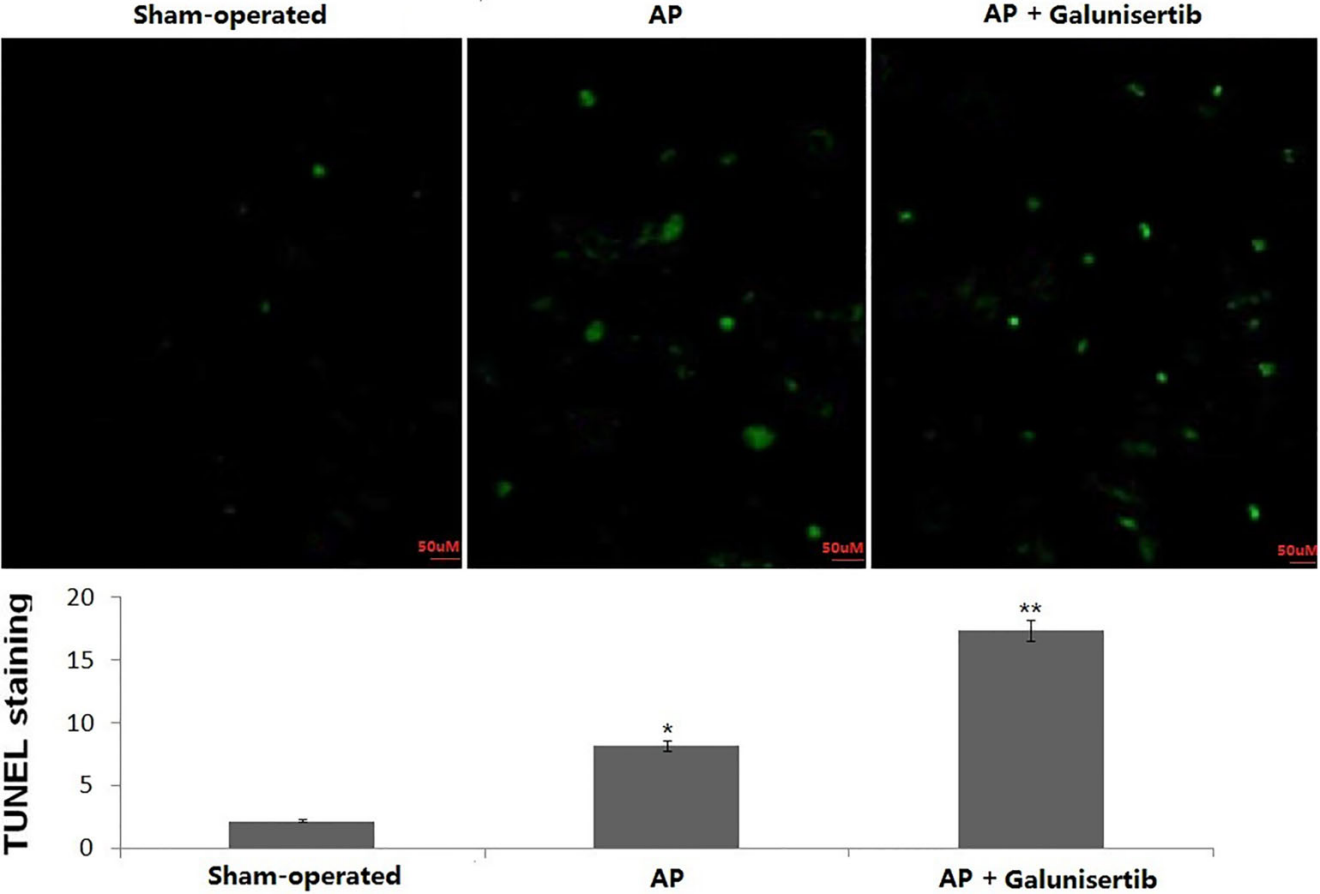
Photographs of TUNEL staining of the pancreatic tissues. The graph shows the average number of fluorescence dots of images from sham-operated, acute pancreatitis (AP), and AP+galunisertib treatment groups. Results are reported as means±SD. *P<0.05, AP+galunisertib *vs* AP and **P<0.01, AP+galunisertib *vs* sham-operated (Student's *t*-test).

Analyzing AP apoptosis *in situ* by TUNEL staining revealed that cell apoptosis was significantly increased in the galunisertib-treated group (16.38±2.26) than in the AP group (8.14±1.27) and sham-operated group (1.82±0.73) (Figure 2). The data suggests that galunisertib can effectively induce acinar cell apoptosis in AP (Figure 2).

### Galunisertib inhibited activation of TGF-β1 signals

**Figure 3:**
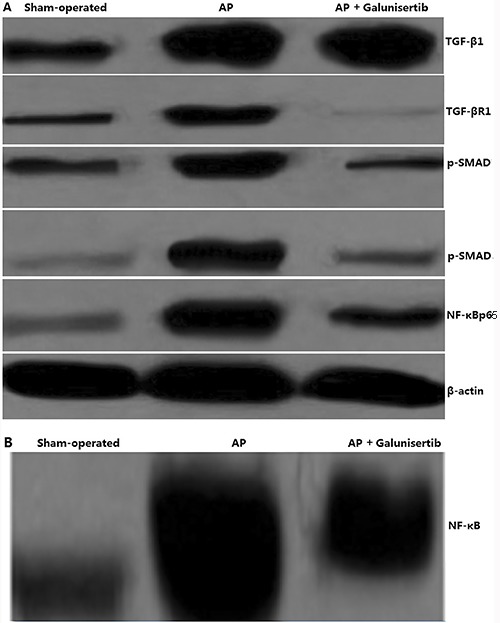
Effect of galunisertib on TGF-β1 signal expression and NF-κB activity. Rats were sham-operated, acute pancreatitis-induced (AP) or AP+galunisertib 2 h after the induction of AP. After 48 h, pancreatic tissues were collected. *A*, Western blot assay for TGF-β1, TGF-βR1, p-Smad2/3 and NF-κBp65 expression. *B*, EMSA assay for NF-κB activity.

In the pancreas of the rats with AP, TGF-β1 was overexpressed compared to the sham-operated group as analyzed by western blotting. To assess the modulation of a candidate receptor for TGF-β signaling, TGF-βRI protein was detected by western blotting. A significant increase in TGF-βRI protein was found in the pancreatitis-induced group compared to the sham-operated group. Next, modulation of the levels of Smad signaling effectors was investigated. Western blot assay showed that phosphorylated Smad2/3 (p-Smad2/3) was less expressed in the pancreas of the sham-operated group; however, high levels of p-Smad2/3 were observed in the pancreas of the AP-induced group. When the rats with AP were treated with galunisertib, TGF-βRI and p-Smad2/3 proteins were significantly inhibited compared to the sham-operated group (Figure 3A).

### Galunisertib inhibited activation of NF-κB signal

The activation of NF-κB plays an important role in the induction of pro-inflammatory cytokines. In the pancreas of rats with AP, NF-κB was significantly activated (82.4±8.3) compared to the sham-operated group (18.5±3.4) as analyzed by EMSA assay (Figure 3B). In addition, NF-κBp65 (p65) translocation was enhanced by western blotting in the AP group compared to the sham-operated group (Figure 3A). To determine the effects of galunisertib on NF-κB activation, the rats were treated with galunisertib 2 hours after the induction of AP. The results showed that NF-κB activation (37.4±10.3) (Figure 3A) and p65 nuclear translocation (Figure 3B) was significantly inhibited by galunisertib treatment.

### Galunisertib reduced serum amylase, lipase, pro-inflammatory cytokines and MPO levels

Serum AMY and LIP are most commonly regarded as biochemical indicators of AP. Thus, we assessed the development of AP by measuring the serum AMY and LIP levels (Table 1). Compared to the sham-operated group, the serum AMY and LIP levels in AP group were significantly increased. However, pre-treatment with galunisertib significantly reduced the elevation of the serum AMY and LIP levels (Table 1). In addition, pre-treatment with galunisertib reduced AP-induced MPO activity in the pancreas (Table 1).

**Table:**
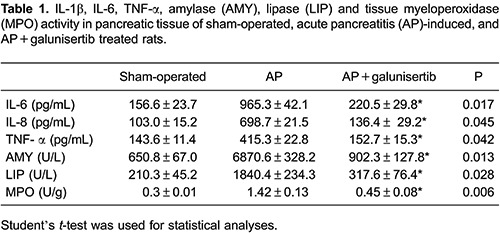

Serum levels of IL-1β, IL-6, and TNF-α in the sham-operated group were significantly lower compared to the AP group, however, pre-treatment with galunisertib significantly reduced the elevation in the serum levels of IL-1β, IL-6, and TNF-α (Table 1).

## Discussion

TGF-β ligands (TGF-β1, TGF-β2, TGF-β3) regulate diverse biological functions (20). TGF-β signaling is initiated when TGF-β ligands engage TGF-β type-I (RI) and type-II (RII) receptors. This induces phosphorylation of the TGF-β receptor kinases (21). The TGF-βRI kinase phosphorylates Smad2 (pSmad2) and Smad3 (pSmad3) resulting in the formation of Smad complexes, which are subsequently translocated into the nucleus to stimulate gene transcription of TGF-β responsive genes (21). Hence, changes in pSmad2 and pSmad3 levels can be used to determine the activity of the TGF-β signaling or inhibitors to this pathway. Recently, TGF-β expression was found to be activated in radiotherapy (22,23), in ischemia and hypoxia stress in the infarcted myocardium (24,25), and in the early phase of AP ([Bibr B08] [Bibr B09] [Bibr B10] [Bibr B11] – 13).

In our TAC-induced rat AP model, TGF-β1, p-SMAD2/3 was activated, and TGF-βRI expression was increased, supporting the above results that TGF-β1 signal is activated in the rat AP model.

The pancreatic enzymes derived from pancreatic acinar cells (AMY, LIP, and the proenzyme trypsinogen) are the cornerstones in the laboratory diagnosis of AP (26). In our TAC-induced rat AP model, serum AMY and LIP, and tissue MPO were significantly increased, indicating that the AP model was successful. Activated macrophages release pro-inflammatory cytokines, such as IL-1β, IL-6, and TNF-α, in response to the local damage of the pancreas (3). As indicated above, local cells contribute to the increase in serum levels of IL-1β, IL-6, and TNF-α in experimental AP (5). These levels correlate with the degree of pancreatic inflammation (27). Our results from TAC-induced AP model agree with the above results.

Among the multitude of inflammatory molecules, a key regulator of cytokine induction is the nuclear transcription factor, NF-κB (28). NF-κB is capable of regulating a variety of inflammatory mediators involved in AP, including TNF-α, IL-1β, and IL-6. It has been confirmed that the activation of NF-κB occurs in pancreatic acinar cells during the initial course of AP and plays a role in the inflammatory response during AP (4). The results from EMSA and western blot analysis revealed that NF-κB was activated and p65 was translocated to the nucleus of the acinar cells following the induction of AP.

Many studies have found that NF-κB pathway is positively regulated by activation of TGF-β1 ([Bibr B29] [Bibr B30]–31). Therefore, targeting TGF-β1 signal, resulting in NF-κB inactivation, might be an effective method for the treatment of AP. In the past, several small molecule inhibitors targeting the TGF-βRI serine/threonine kinase activity have been developed, including galunisertib monohydrate (32), which has been found to inhibit pSMAD2 expression in different tumor models (33,34).

In the present study, we used a TAC-induced rat AP model for investigating the protective effects of galunisertib. Our study showed that treatment with galunisertib inhibited AP-induced TGF-β1, pSMAD2/3, and NF-κB activation, and reduced TGF-βRI expression. Also, galunisertib treatment downregulated inflammation signaling including IL-1β, IL-6, and TNF-α. In addition, galunisertib treatment significantly alleviated edema, hemorrhage, necrosis, and inflammatory responses of TAC-induced experimental AP in rats, and decreased AP markers, AMY, LIP, and MPO activity.

It has recently been found that apoptosis generally limits the inflammatory cascade; the apoptosis rate of acinar cells is inversely related to AP severity (35). Thus, induction of apoptosis in pancreatic acinar cells exerts a protective effect (36), whereas suppression of apoptosis increases the severity of AP (36). Our data demonstrate that cell apoptosis was increased in the acinar cells of AP, after galunisertib treatment apoptotic cells were significantly increased, supporting the idea that increased cell apoptosis protects acinar cells from injury.

We have found here that galunisertib administration inhibited TGF-β signal and reduced NF-κB translocation and release of pro-inflammatory factors in TAC-induced AP rat model. Our study indicates that TGF-β plays a pivotal role in the cellular mechanisms underlying experimental pancreatitis resulting in a severe inflammatory response. Our data demonstrate that galunisertib attenuates the severity of TAC-induced experimental AP in rats by inducing apoptosis in acinar cells, and inhibiting the activation of TGF-β and NF-κB signal, and suppressing the secretion of pro-inflammatory cytokines. For the first time, we show that the anti-tumor agent galunisertib may have a therapeutic potential in reducing inflammatory defense mechanisms and the injury resulting from AP.
